# The translational potential of salvinorin A: systematic review and meta-analysis of preclinical studies

**DOI:** 10.1038/s41398-025-03638-3

**Published:** 2025-10-10

**Authors:** Wolfgang Emanuel Zürrer, Lionel Wettstein, Helena D. Aicher, Milan Scheidegger, Benjamin Victor Ineichen

**Affiliations:** 1 Center for Reproducible Science and Research Synthesis, University of Zurich, Zurich, Switzerland; 2 Neuroscience Center Zurich, University of Zurich and ETH Zurich, Zurich, Switzerland; 3 Department of Chemistry and Applied Biosciences, ETH Zurich, Zurich, Switzerland; 4 Department of Adult Psychiatry and Psychotherapy, Psychiatric University Clinic Zurich and University of Zurich, Zurich, Switzerland; 5 Department of Clinical Research, University of Bern, Bern, Switzerland; 6 Department of Psychiatry, Division of Medicine, University of Basel, Basel, Switzerland

**Keywords:** Pharmacology, Neuroscience, Psychiatric disorders

## Abstract

**Background:**

Salvinorin A, the main psychoactive compound of Salvia divinorum, is a potent and selective kappa opioid receptor agonist. While human clinical trials remain limited, animal studies suggest potential therapeutic applications in neurological and psychiatric disorders. This systematic review and meta-analysis aims to synthesize these preclinical findings, addressing three questions: (1) What is the therapeutic potential of salvinorin A in animal models of neurological and psychiatric disorders? (2) What are its toxic effects on behaviour, cognition, and physiological function? (3) What are its pharmacokinetic characteristics?

**Methods:**

A systematic search of Medline, Web of Science, and EMBASE for studies published up to June 28, 2024, identified 1718 publications, of which 82 were included in the qualitative synthesis and 10 in the meta-analysis.

**Results:**

Salvinorin A has been tested in animal models of pain, cerebrovascular insults, addiction, and depression. It exhibited anti-nociceptive, anti-inflammatory, neuroprotective, and anti-addictive effects. Findings on depression were inconsistent, with both antidepressant and depressogenic outcomes reported. Toxicity data indicate anxiogenic effects and motor and cognitive impairment, with minimal impact on vital parameters. Applied doses ranged from 0.1–10 mg/kg, with lower doses in stroke models. Pharmacokinetic data show rapid onset, fast peak, and a half-life of approximately one hour. Sixteen structurally distinct salvinorin A analogues were identified with potentially improved safety and pharmacokinetic profiles.

**Conclusion:**

Our findings support the therapeutic potential of salvinorin A for pain, addiction, and stroke, though its side effect profile may limit clinical application. The development of novel analogues could address these challenges.

## Introduction

Psychedelics have re-emerged as a promising frontier in neurological and psychiatric disease treatment, showing therapeutic potential for, among others, mood disorders [1, 2], substance use disorders [3], post-traumatic stress disorder [4], and migraine [5]. While classical psychedelics primarily act as serotonin 5-HT2A receptor agonists, there is growing interest in psychedelics with distinct mechanisms of action. In this review, we use the term ‘atypical psychedelic’ to refer to compounds that produce profound alterations in consciousness but act through non-serotonergic mechanisms. One such example is salvinorin A, the main active ingredient of Salvia divinorum, a psychedelic plant traditionally used by the Mazatec people of Oaxaca, Mexico [6, 7]. Unlike classical psychedelics, salvinorin A is a potent and selective kappa opioid receptor (KOR) agonist [8]. This pharmacological distinction provides new possibilities for therapeutic exploration in conditions that may not respond to existing treatments or serotonergic psychedelics. Preclinical studies suggest that salvinorin A has potential applications in treating addiction [9, 10], depression [11, 12], pain [13, 14], and stroke [15, 16]. However, clinical translation is challenged by its rapid metabolism and concerns over adverse effects, including anxiety, motor impairment, aversion, and psychotomimetic experiences with intense dissociative effects at higher doses [17–20].

Despite the potential of salvinorin A, the body of preclinical evidence regarding its therapeutic and toxicological profile remains fragmented and inconsistent, with no comprehensive synthesis to evaluate its benefits and risks. This comes at the cost of a potentially promising treatment candidate for neuropsychiatric disorders not being translated to clinical practice. Furthermore, synthesizing existing findings may provide insights to develop additional novel therapeutics targeting the kappa opioid receptor, a pathway largely underexplored in clinical settings [21].

The objective of this systematic review is to synthesize the preclinical evidence on salvinorin A’s application in neurological and psychiatric diseases. Specifically, we aim to address the following questions: (1) In which animal models of neurological and psychiatric diseases has salvinorin A been tested and what were the observed effects? (2) Does salvinorin A supplementation to animals cause adverse effects? (3) What are the pharmacokinetic properties of salvinorin A in animals?

## Methods

### Protocol and reporting standards

We pre-registered the study protocol in the Open Science Framework (OSF, https://osf.io/862yz/) and used the Preferred Reporting Items for Systematic Reviews and Meta-Analysis (PRISMA) 2020 Guidelines for reporting [22].

### Search strategy

We searched for studies in Medline via PubMed, Web of Science, and EMBASE via the Ovid interface from inception to June 28, 2024. See Table S1 for the search string in each of these databases. Reference lists of eligible publications were screened for additional papers.

### Inclusion and exclusion criteria

#### Inclusion criteria

Any original study that investigated the effects of salvinorin A or an analogue of salvinorin A in vertebrate animal models of neurological or psychiatric diseases.

#### Exclusion criteria

Studies that assessed salvinorin A in vitro or in silico, studies focusing on non-psychedelic compounds of Salvia divinorum (e.g., salvinorin B or KOR-agonists that are not derived from salvinorin A), and studies involving other psychedelic substances. Grey literature (e.g., conference abstracts, book chapters, patents) and (systematic) reviews were excluded but retained as sources for additional references.

### Study selection and data extraction

Two reviewers (WEZ and BVI) screened titles and abstracts of studies for their relevance in the web-based application Rayyan [23], followed by full-text screening. Subsequently, we extracted the following data: title, authors, publication year, study country, substances tested, animal species and sex, number of subjects, disease model, outcome measures for neurological/psychiatric disease models, applied doses, mode of drug application, effects of substances including toxic effects, and pharmacokinetics.

### Critical appraisal of included studies

Risk of bias was assessed using a pre-defined 6-item checklist: (i.) Reporting of randomization, (ii.) Reporting of blinding, (iii.) Reporting of an animal welfare statement, (iv.) Statement of a potential conflict of interest, (v.) Sample size calculations provided, and (vi.) In accordance with the ARRIVE guidelines [24].

### Data synthesis and analysis

Findings were summarized in a narrative fashion complemented by descriptive statistics of extracted parameters. Toxicity assessments focused on physiological measures (e.g., heart rate, blood pressure) alongside neurobehavioral evaluations, including motor functioning, cognitive functioning, sedation, or signs of anxiety. Additionally, studies examining the behavioural response to salvinorin A using a conditioned place preference or condition taste aversion paradigm were summarized.

For the quantitative synthesis, we conducted meta-analyses on outcomes reported 3 or more times. If multiple doses were provided, the dose showing the highest effect was chosen for quantitative analysis. As primary effect measure, Hedges’ g standardised mean difference (SMD) was used which was pooled to obtain an overall SMD and 95% confidence intervals, using the R package metafor for the meta-analysis. A random-effects model was fitted to the data [25]. The amount of heterogeneity, i.e., τ2, was estimated using the DerSimonian-Laird estimator. The Q-test for heterogeneity and the I2 statistic were calculated. We conducted all statistical analyses in the R programming environment (version 4.2.2). We considered a two-tailed p-value less than 0.05 statistically significant.

### Publication bias

We did not assess publication bias.

## Results

### Eligible publications

Our search yielded 1718 unique studies, with 145 studies identified for full-text review (Fig. S1). Of these, 82 studies were included in the descriptive synthesis and 10 studies in the quantitative analysis (meta-analysis). Notably, 11 studies focused exclusively on analogues of salvinorin A.

### General study characteristics

**Fig. 1:**
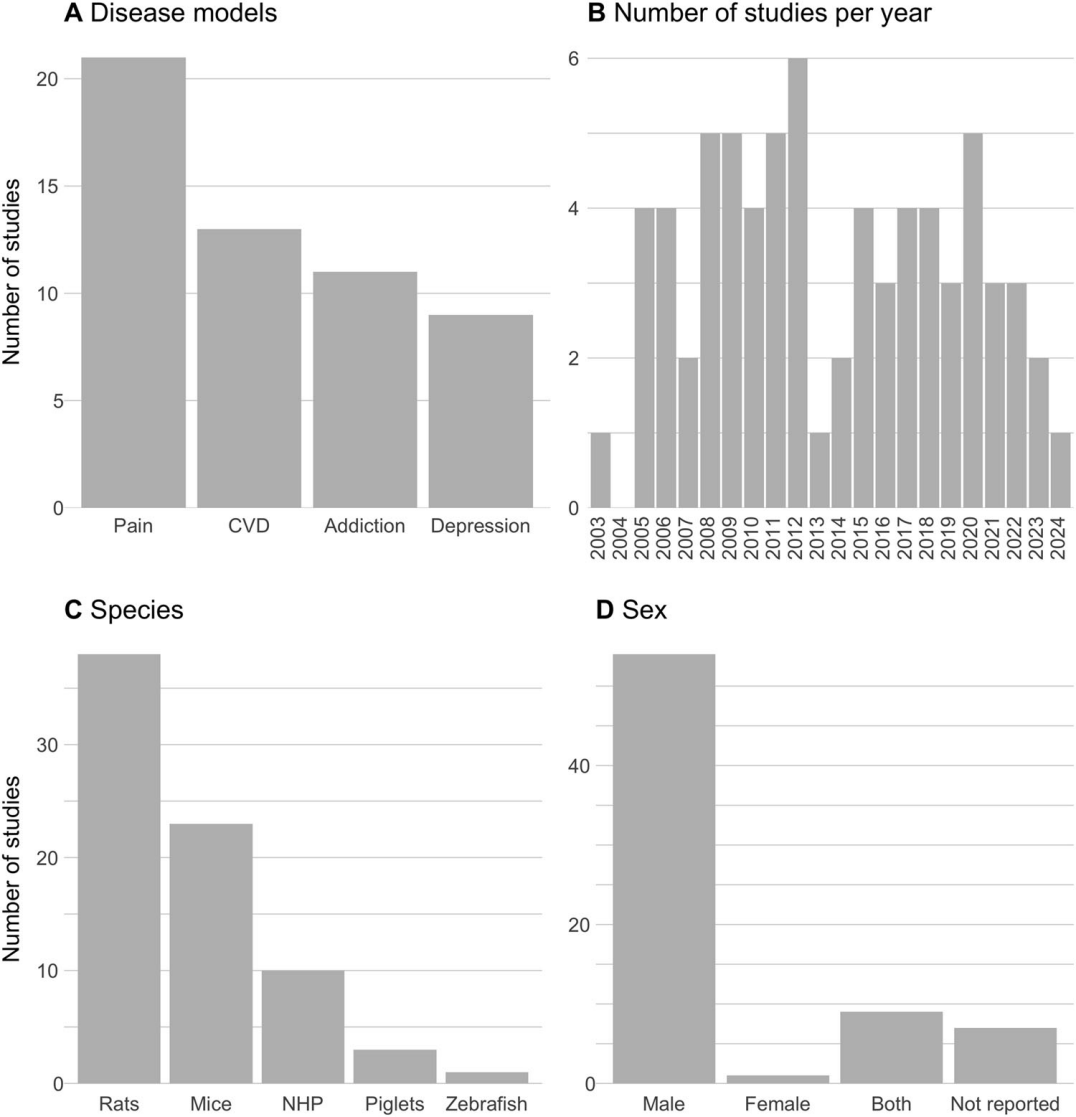
Study characteristics. Number of studies per study characteristic: Used disease model **A**, year of publication **B**, species **C**, and sexes **D** in animal studies assessing the therapeutic potential of salvinorin A. Abbreviations: CVD, cerebrovascular diseases; NHP, non-human primates.

Most studies focused on pain (30%, n = 21), followed by cerebrovascular diseases (18%, n = 13), addiction (16%, n = 11), and depression (13%, n = 9) (Fig. 1A). Twenty-nine studies reported on toxicity and nine studies explored behavioural responses to salvinorin A.

The timeline of the included studies spanned from 2003 to 2024 (Fig. 1B).

Regarding animal species, most studies utilized rats (54%, n = 38), followed by mice (31%, n = 23), non-human primates (14%, n = 10), piglets (4%, n = 3), and zebrafish (1%, n = 1) (Fig. 1C).

The median number of animals used per study was 48, resulting in approximately 3456 animals being used to test the effects of salvinorin A in neurological and psychiatric disease models. Animal sex was reported in 90% of studies (n = 64), 76% only utilized male animals (n = 54) (Fig. 1D).

Studies originated from 7 countries, with the majority conducted in the United States (58%, n = 41).

#### Risk of bias assessment

Most studies reported an animal welfare statement (93%, n = 76). 55% of studies reported the presence or absence of a potential conflict of interest (n = 45). Only 32% of animal studies reported blinding, and the same percentage reported randomization (n = 26 each). Additionally, only one study reported an a priori sample size and three studies reported in accordance with ARRIVE guidelines (Table S2).

### Pharmacokinetics

**Fig. 2:**
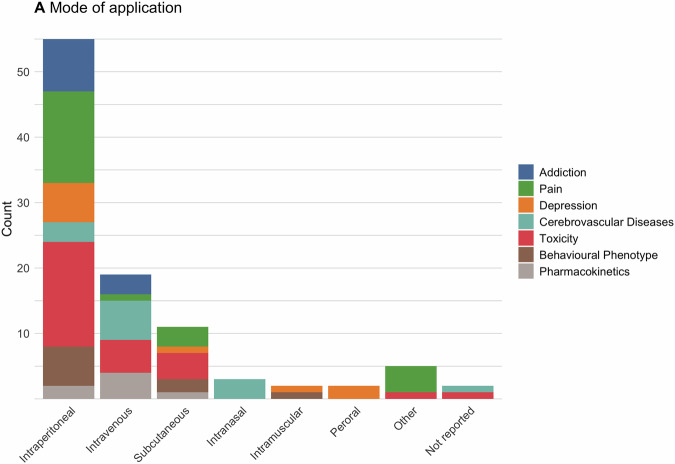
Pharmacokinetics: Mode of application. Frequency of application modes used in experiments assessing the therapeutic potential of salvinorin A. Each mode was counted separately for every assessment category conducted within a study. Two studies that did not report doses were excluded from the analysis.

Intraperitoneal administration was the primary mode of application in most studies investigating salvinorin A (78%, n = 55), followed by intravenous (27%, n = 19) and subcutaneous administration (16%, n = 11) (Fig. 2). Less common routes included intranasal (4%, n = 3), intramuscular (3%, n = 2), oral (3%, n = 2), and a variety of alternative approaches such as intraplantar [26], intrathecal [13], intracerebroventricular administration [27], and direct injections into cerebral grey matter [28]. Some studies used more than one mode of application.

**Fig. 3:**
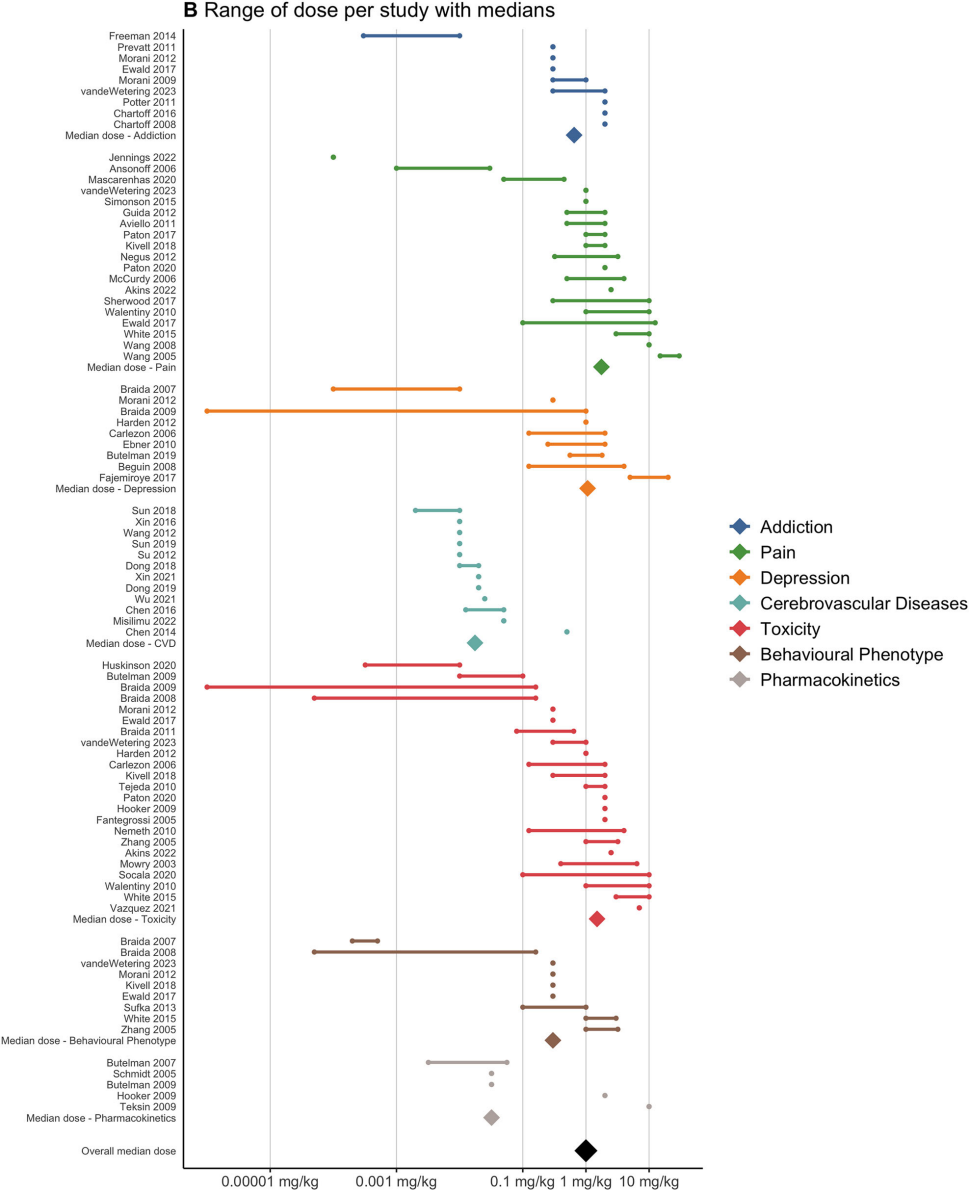
Pharmacokinetics: Range of doses. Range of doses used in studies assessing the therapeutic potential of salvinorin A. Each range is displayed separately for every assessment category within a study. Nine studies that did not report doses were excluded from this analysis.

Doses ranged between 0.1–10 mg/kg body weight across most experiments, with notable variation across disease categories (Fig. 3). For instance, studies focusing on cerebrovascular diseases tended to employ 2 orders of magnitude lower doses.

Salvinorin A’s pharmacokinetic profile was characterized by rapid onset and fast peak concentrations [29–33]. In a study using positron emission tomography (PET) imaging in baboons, salvinorin A reached maximum brain concentrations within 40 s of intravenous administration [33]. Even faster brain uptake was observed in rats, with peak activity occurring within 20 s [31]. After intraperitoneal dosing in rats, maximum plasma and brain concentrations were reached at 15 and 10 min, respectively [32]. Brain uptake corresponded to 3.3% of the administered dose for intravenous administration [33] and 5% for intraperitoneal administration [32]. The brain half-life of salvinorin A ranged from 3 min in rats following intraperitoneal administration and 8 min in baboons after intravenous administration (both measured using positron emission tomography imaging) [31, 33], to 36 min in rats after intraperitoneal administration (measured via liquid chromatography–mass spectrometry) [32]. Elimination half-life in the plasma was reported as 57 min following intravenous administration in rhesus monkeys [34] and 75 min after intraperitoneal dosing in rats [32].

### Addiction

Eleven studies evaluated the effects of salvinorin A on addiction-related behaviours, focusing primarily on cocaine (n = 9), with additional studies investigating remifentanil (n = 1) and oxycodone (n = 2) (Table S3). Salvinorin A demonstrated anti-addictive properties in nine studies. In rat cocaine studies, it attenuated drug-seeking behaviour and reinstatement in self-administration paradigms [35–39]. Repeated administration of salvinorin A reduced cocaine’s reward-potentiating effects as measured by intracranial self-stimulation [40]. However, two studies reported mixed effects, with salvinorin A either potentiating or reducing behavioural and molecular responses depending on timing [41, 42]. In studies involving opioids, salvinorin A reduced oxycodone self-administration in progressive ratio paradigms and decreased choice for remifentanil in rhesus monkeys using concurrent-choice schedules [9, 10, 43].

### Pain

Twenty-one studies investigated the effect of salvinorin A in diverse models of acute, inflammatory, and neuropathic pain (Table S4). Fifteen studies showed antinociceptive effects, while six reported anti-inflammatory properties. Salvinorin A consistently increased pain thresholds in thermal and mechanical assays, such as tail flick, hot plate, and formalin tests [13, 14, 27, 35, 38, 44–49]. Anti-inflammatory effects included the reduction of lipopolysaccharide (LPS)- and carrageenan-induced paw oedema, decreased glial activation, and suppression of inflammatory markers in the formalin test [44–46, 50–52]. Additionally, salvinorin A reduced mechanical and heat allodynia in two studies [26, 51]. In contrast, four studies reported minimal or no analgesic effects in thermal or writhing pain tests [53–56].

**Fig. 4:**
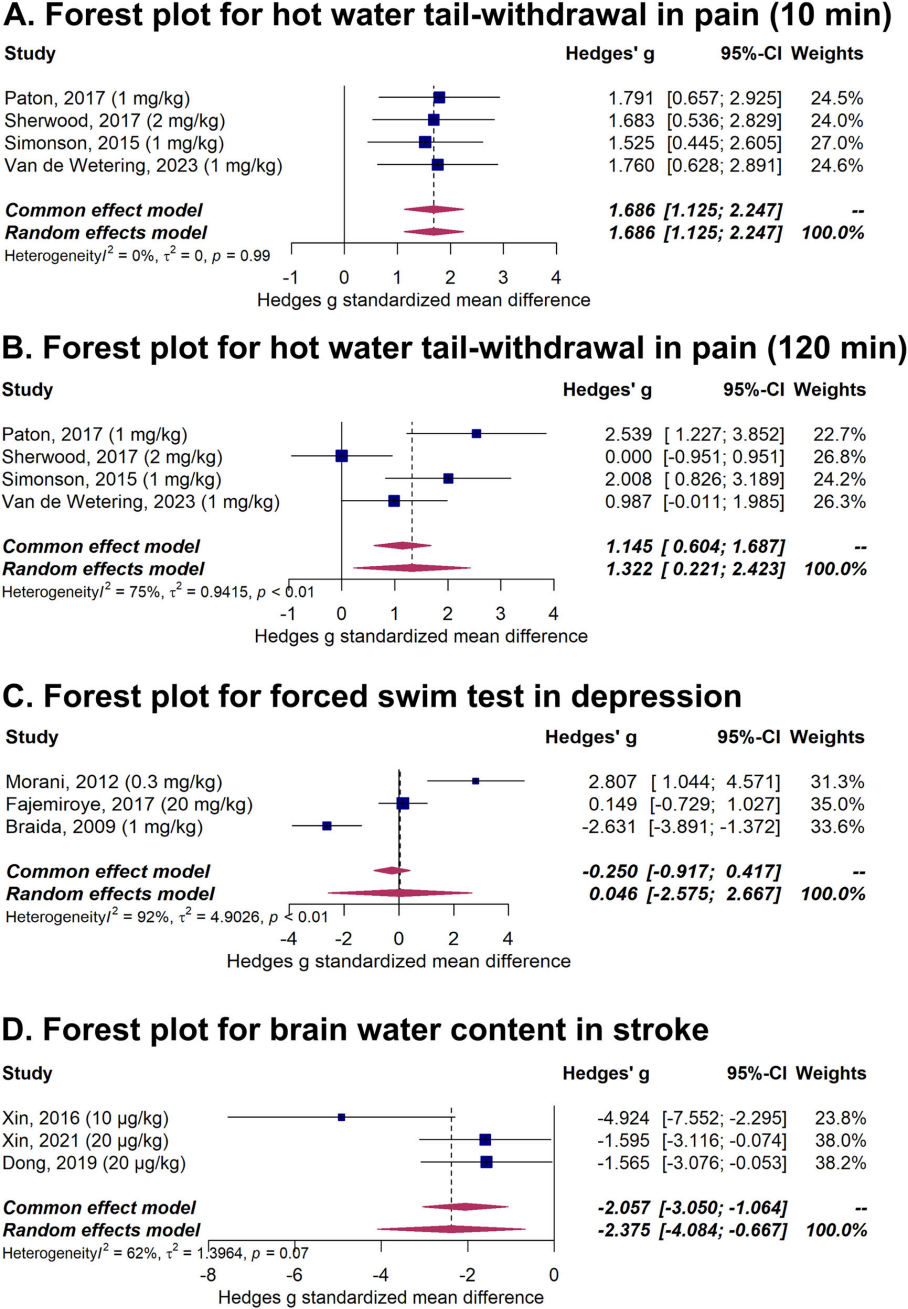
Meta-analysis on outcome measures of neurological and psychiatric disease models. Forest plots showing the pooled standardized mean differences (Hedges’ g) for the effects of salvinorin A on **A** pain threshold at 10 min (Hedges’ g: 1.69 [95%-CI: 1.13–2.25]; I^2^ = 0%), and **B** at 120 min (Hedges’ g: 1.32 [95%-CI: 0.22–2.42]; I^2^ = 75%), both demonstrating an increase in pain tolerance. **C** Salvinorin A did not significantly affect depressive behaviour in the forced swim test (Hedges’ g: 0.05 [95%-CI: −2.58–2.67]; I^2^ = 92%). **D** Salvinorin A reduced brain water content (edema) (Hedges’ g: −2.38 [95%-CI: −4.09–−0.67]; I^2^ = 62%), indicating a neuroprotective effect. A random-effects model was used for all analyses.

Pooling four studies in a meta-analysis showed that salvinorin A increased the pain threshold at both 10 min (Fig. 4A, random effects model, Hedges’ g: 1.69 [95%-CI: 1.13–2.25]; I^2^ = 0%) and 120 min (Fig. 4B, random effects model, Hedges’ g: 1.32 [95%-CI: 0.22–2.42]; I^2^ = 75%) [38, 45, 47, 52].

### Depression

Nine studies investigated the effects of salvinorin A on depressive-like behaviours in animal models (Table S5). Of these, two studies reported an antidepressant effect, five studies observed depressogenic effects, one study noted mixed effects, and one study found no effect. Salvinorin A elevated intracranial self-stimulation thresholds in multiple studies and decreased dopamine release in the nucleus accumbens [57–59]. Conflicting results were reported in the forced swim test and tail suspension test, with two studies observing increased immobility upon salvinorin A administration indicative of depressogenic effects [37, 58], one study reporting reduced immobility suggesting antidepressant outcomes [11], and one study finding no alterations [60]. Importantly, in studies where salvinorin A produced depressive-like effects in the forced swim test, the respective doses did not impair spontaneous locomotion in the open field test [37, 58]. In one study, chronic administration of salvinorin A reversed anhedonia in the sucrose preference test following chronic mild stress [12]. In another study using zebrafish, dose-dependent effects of salvinorin A on depression were reported, with stimulating effects observed at lower doses and depressive effects at higher doses [61]. The timing of behavioral assessments varied across studies: Most examined effects shortly after a single administration of salvinorin A [11, 37, 61, 62], whereas investigations into chronic administration were scarce [12].

Pooling three studies in a meta-analysis showed that salvinorin A did overall not affect depressive behaviour as measured by the forced swim test (Fig. 4C, random effects model, Hedges’ g: 0.05 [95%-CI: −2.58–2.67]) [11, 37, 60]. However, there was substantial overall heterogeneity between the studies (I^2^ = 92%).

### Cerebrovascular diseases

Thirteen studies assessed the putative neuroprotective effects of salvinorin A in animal models of cerebrovascular diseases, including ischemic stroke, hypoxia, and subarachnoid haemorrhage (Table S6). Salvinorin A consistently demonstrated neuroprotective properties both in acute (within hours and days) and chronic (up to several weeks) assessments, with reductions in infarct size, neurological deficits, and blood-brain barrier permeability [15, 63–69]. It decreased inflammation and apoptosis and improved sensory, motor, and cognitive outcomes [16, 63, 64, 66, 68]. Vasoprotective effects were also observed, including the preservation of pial artery autoregulation in response to hypercapnia and hypotension [64, 70–72]. In neonatal mice exposed to hypoxia, salvinorin A improved survival rates and accelerated neurodevelopmental milestones, although no long-term effects were observed [73]. Benefits on cerebral vasospasm and early brain injury were noted following subarachnoid hemorrhage [16, 67].

Pooling three studies in a meta-analysis showed that salvinorin A reduced brain water content, i.e., edema (Fig. 4D, random effects model, Hedges’ g: −2.38 [95%-CI: −4.09–−0.67]) [65, 68, 69]. However, there was substantial overall heterogeneity between the studies (I^2^ = 62%).

### Toxicity and adverse events

In terms of physiological effects, studies in rodents and baboons reported no significant changes in cardiac conduction, blood pressure, heart rate, pO2, body temperature [33, 74]. Chronic administration at doses up to 6.4 mg/kg body weight daily for 14 days also showed no alterations in organ histology [74]. Similarly, no treatment-related effects were observed on reproductive functions, seizure thresholds, or sensorimotor gating [12, 75, 76].

Anxiety as a potential adverse effect of salvinorin A was assessed in nine studies: Seven studies found an anxiogenic effect of salvinorin A, using the elevated plus maze, elevated zero maze, light-dark test, defensive burying behaviour test, and defensive and tonic immobility duration [28, 35, 38, 44, 46, 77, 78]. Two studies reported anxiolytic-like effects in the elevated plus maze test, without a clear dose-dependency [11, 56].

Motor function upon salvinorin A administration was assessed in sixteen studies: Eight studies identified impaired locomotion following salvinorin A administration [31, 44, 46, 48, 49, 79–81], while eight other studies found no such impairment on motor activity or coordination [11, 12, 37, 56, 58, 75, 82, 83].

Cognitive function upon salvinorin A administration was assessed in four studies: Two studies found impairments: Salvinorin A disrupted spatial long-term memory, episodic memory, and attention in tasks like latent inhibition and the 5-choice serial reaction time task [83, 84]. In contrast, two studies observed no negative effects on memory and learning in the novel object recognition task [38, 44].

Sedation was assessed in three studies, with all demonstrating sedative-like properties [29, 79, 80]. Dose-dependent sedation was observed, with effects peaking shortly after administration and dissipating within 30 min after intravenous administration of salvinorin A [29].

Rewarding or aversive effects of salvinorin A were assessed in nine studies using conditioned place preference (CPP) and conditioned place aversion (CPA) paradigms [35, 37, 38, 44, 49, 61, 81, 82, 85]. Several studies reported consistent CPA across all tested doses [35, 38, 81], while one study observed no significant aversion or preference at specific doses [49]. In two studies low to moderate doses (0.1–40 μg/kg) produced CPP, indicating potential rewarding properties, while higher doses (≥80 μg/kg) induced CPA [61, 82]. In addition, salvinorin A did not elicit conditioned taste aversion in two studies [37, 44]. In a study assessing the abuse potential, salvinorin A did not support stable self-administration behaviour in rats at doses of 0.5 or 1.0 μg/kg/infusion, suggesting a low potential for abuse [86].

### Analogues of salvinorin A

**Fig. 5:**
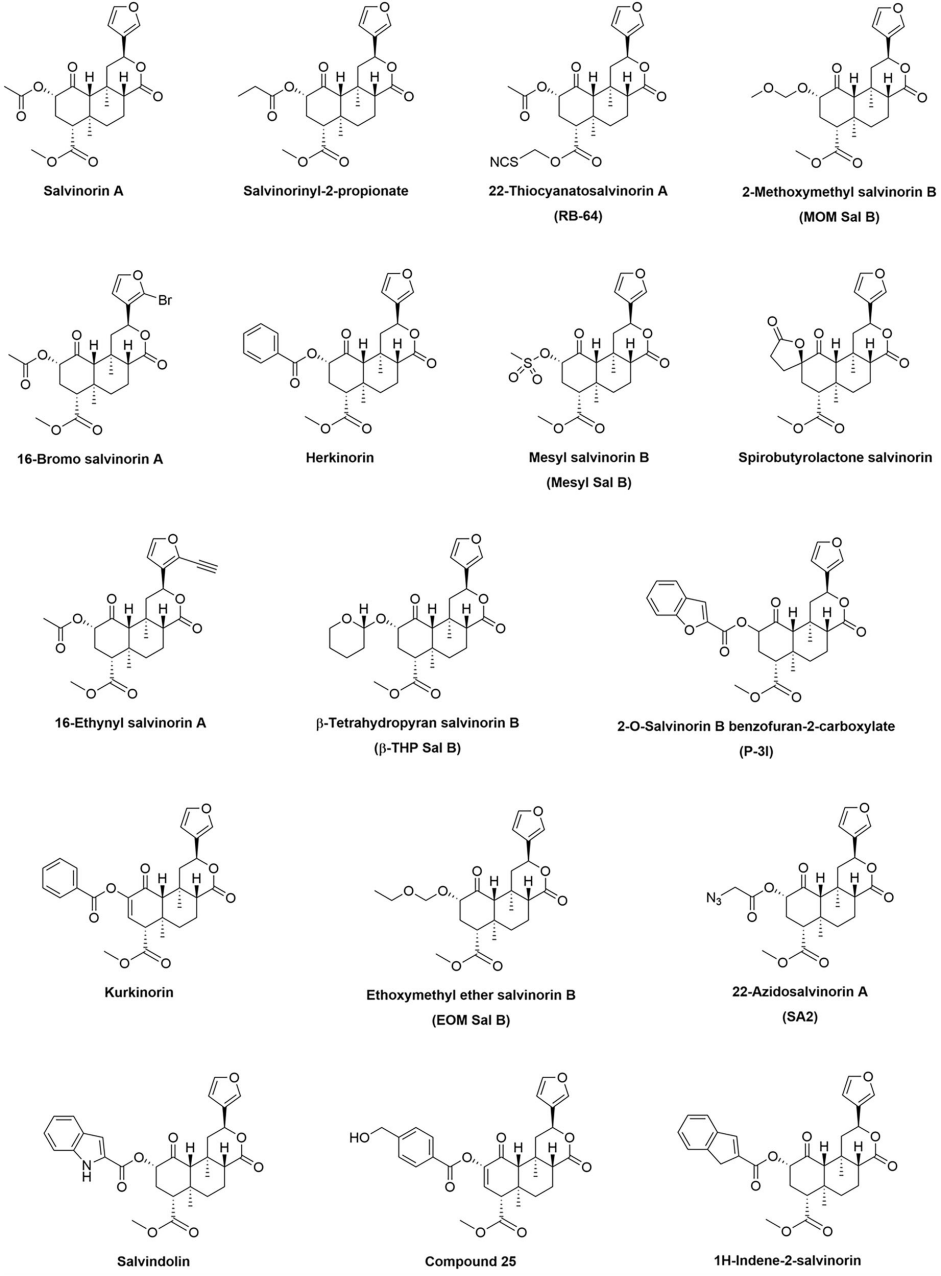
Chemical structures of salvinorin A and its analogues. Structures of salvinorin A and 16 analogues investigated for therapeutic potential in neurological and psychiatric disorders.

We identified 16 molecularly distinct analogues of salvinorin A with potential clinical applications in various neurological and psychiatric disorders (Table 1, Fig. 5). These analogues have gained attention in recent years (Fig. S2), primarily due to their optimized pharmacokinetic profiles and a more favourable side effect profile compared to the parent compound. Interestingly, one analogue, ethoxymethyl ether salvinorin B, demonstrated therapeutic efficacy by promoting remyelination in an animal model of multiple sclerosis – an indication that has not been previously explored for salvinorin A [87].

**Table 1:** Analogues of salvinorin A.

Salvinorin A analogue (n = 16)	Clinical application	Comparison to salvinorin A
Salvinorinyl-2-propionate	Pain [27]	Lower efficacy in antinociception.
22-Thiocyanatosalvinorin A (RB-64)	Pain [49]	Improved side effect profile inducing less sedation or anhedonia-like effects.
2-Methoxymethyl salvinorin B (MOM Sal B)	Addiction [99], Pain [53]	Higher potency and longer duration of action. Improved side effect profile inducing less sedation.
16-Bromo salvinorin A	Addiction [38, 100], Pain [38, 46, 101]	Longer duration of action and improved side effect profile inducing less motor incoordination, cognitive dysfunction, anxiety, and depressive-like behaviour.
Herkinorin	Cerebrovascular diseases [102, 103], Pain [104]	Primarily acts peripherally on the mu-opioid receptor.
Mesyl salvinorin B (Mesyl Sal B)	Addiction [44, 47], Pain [47]	Longer duration of action and improved side effect profile inducing less sedation.
Spirobutyrolactone salvinorin	Pain [52]	Higher metabolic resistance.
16-Ethynyl salvinorin A	Addiction [100], Pain [46, 101]	Longer duration of action and higher efficacy. Improved side effect profile inducing less motor incoordination, anxiety, and depressive-like behaviour.
β-Tetrahydropyran salvinorin B (β-THP Sal B)	Addiction [35, 39], Pain [45]	Comparable efficacy in reducing cocaine-induced drug-seeking. Improved side effect profile inducing less motor incoordination, anxiety or depressive-like behaviour.
2-O-Salvinorin B benzofuran-2-carboxylate (P-3l)	Anxiety [105], Pain [105]	Anxiolytic-like effect.
Kurkinorin	Pain [106]	Potent and selective mu opioid receptor agonist.
Ethoxymethyl ether salvinorin B (EOM Sal B)	Addiction [35], Multiple Sclerosis [87], Pain [101]	Higher potency and efficacy in animal models of demyelinating diseases. Improved side effect profile inducing less motor incoordination, anxiety or depressive- and aversive-like effects.
22-Azidosalvinorin A (SA2)	Depression [60]	Antidepressant effect.
Salvindolin	Depression [107], Pain [107]	Dual agonism towards kappa- and mu-opioid receptors. Good oral bioavailability. Improved side effect profile inducing less motor incoordination and an antidepressant effect.
Compound 25	Pain [108]	Primarily acts on the mu-opioid receptor.
1H-Indene-2-salvinorin (Compound 2)	Pain [56]	Dual agonism on kappa- and mu-opioid receptors. Anxiolytic-like effect

## Discussion

### Main findings

This study systematically assessed the translational potential of salvinorin A for neuropsychiatric diseases. Salvinorin A has been tested in various animal models, primarily for pain, cerebrovascular insults, addiction, and depression. It showed anti-nociceptive, anti-inflammatory, and antiallodynic effects in pain models, neuroprotection in cerebrovascular diseases, and anti-addictive properties. However, its effects on models of depression were inconsistent, with studies reporting antidepressant-like effects and others indicating depressogenic outcomes. Neurobehavioral and toxicity/adverse events data suggest that salvinorin A exerts anxiogenic effects and impairs motor and cognitive function but has limited impact on vital parameters. It was administered at doses ranging from 0.1–10 mg/kg (mostly intraperitoneal, intravenous, or subcutaneous), with stroke studies using lower doses. Pharmacokinetics differ by administration route, showing onset and peak concentrations within seconds to minutes, a brain half-life of 3–36 min, and plasma elimination around an hour. We identified 16 molecularly distinct salvinorin A analogues with potentially improved side effect profiles.

### Findings in the context of existing evidence

Animal studies have evaluated salvinorin A in models of pain, addiction, depression, and cerebrovascular diseases, conditions prevalent and thus relevant to human health. Preclinical findings suggest salvinorin A’s potential in these areas, particularly its relatively consistent anti-nociceptive, neuroprotective, and anti-addictive properties [21]. In humans, kappa opioid receptor agonists have been explored for pain relief and addiction treatment [88], but salvinorin A’s psychoactive effects and short duration of action have limited its clinical use [89]. Evidence from small clinical trials indicates that inhaled salvinorin A induces profound but transient perceptual alterations in humans, including strong dissociative effects, with little evidence of reinforcing effects [20, 90], differentiating it from classic opioids [21]. However, no clinical trials have evaluated its therapeutic potential for these conditions. While salvinorin A has been considered for depression treatment [91], preclinical data are inconsistent, with several studies reporting depressogenic effects.

Salvinorin A is structurally distinct from other opioids, lacking the positively charged nitrogen atom traditionally required for opioid receptor interaction. It is highly potent, with CNS effects observed at 200–500 μg in humans [92, 93]. Interestingly, salvinorin A appears to exert neuroprotective effects in stroke at substantially lower doses compared to those used in models of depression, pain, and addiction, where doses are often 100 times higher [15]. The classical psychedelic N,N-dimethyltryptamine (DMT) has also been tested for stroke and salvinorin A might be a potential neuroprotective alternative [94]. However, it has poor bioavailability due to rapid enzymatic degradation and P-glycoprotein-mediated efflux at the blood-brain barrier [95]. Thus, oral administration, including buccal and sublingual routes are limited by saliva degradation. Intravenous formulations allow dose control but often require pharmaceutically unsuitable solvents. Thus, alternative delivery methods such as intranasal sprays may enhance bioavailability [96]. The variation in reported brain half-life across studies may reflect differences in analytical methods, with shorter values from positron emission tomography imaging and longer values from liquid chromatography–mass spectrometry.

We identified 16 molecular analogues of salvinorin A, with potential advantages over the parent compound [97]. Some analogues exhibit improved pharmacokinetics, including longer half-life and enhanced brain penetration. Others demonstrate altered receptor selectivity, which could modulate therapeutic and adverse effects. Notably, some derivatives retain neuroprotective properties while reducing psychoactive side effects, broadening their potential clinical applications. And unlike classical psychedelics, salvinorin A does not appear to affect vital cardiovascular parameters, which could make it a preferable option for patients with cardiovascular conditions [98].

Salvinorin A meets key criteria for further translational assessment in humans, including a relatively well-understood mechanism of action, dose-response studies with clinically relevant treatment durations, and multiple assessed outcome measures. However, barriers to translation remain: Modeling psychiatric diseases in rodents is inherently limited due to their complex psychological phenotypes. Additionally, as in most experimental fields, there is a sex bias, with salvinorin A predominantly tested in young, male rodents without comorbidities, which may limit the generalizability of findings across sexes and genders in humans.

### Limitations

Our study should be interpreted with certain limitations in mind. First, the pooled data exhibited substantial heterogeneity in methodology and reporting, increasing the risk of bias in synthesis. Second, the broad scope of this review across multiple diseases prevents a detailed discussion of administration routes and doses for each included study in the body of the manuscript. However, this information is available in the (supplementary) tables for interested readers.

### Strengths

This study is the most comprehensive and rigorous synthesis to date of animal studies on the therapeutic potential of salvinorin A. It covers a wide range of aspects, including its application, toxicity, adverse events, pharmacokinetics, and efficacy.

### Conclusions

Despite human reports of challenging subjective experiences, our review of preclinical studies suggests that salvinorin A might be a promising candidate for treating pain, addiction, and stroke, as an atypical psychedelic-based approach. This potential is supported by a generally favourable physiological safety profile. However, its unpredictable dissociative mental effects necessitate strictly supervised administration to mitigate harm. The development of optimized derivatives, dosing protocols, or formulations with prolonged activity offers a pathway to address these limitations. Further human studies and risk-benefit assessments are needed to clarify whether salvinorin A and these analogues offer therapeutic efficacy with acceptable safety in psychiatric or neurological populations.

## Availability of data, code and materials

The dataset and code supporting the conclusions of this article are available on the Open Science Framework (OSF, https://osf.io/862yz/).

## Supplementary information

Filled PRISMA reporting checklist 2020

Supplementary data
