# Supplementary material for: The translational potential of salvinorin A: systematic review and meta-analysis of preclinical studies
Source: Transl Psychiatry. 2025 Oct 10;15:401. doi: 10.1038/s41398-025-03638-3 (PMC12514287; doi:10.1038/s41398-025-03638-3)
Supplement: Supplementary file 2 — Supplementary data [file 41398_2025_3638_MOESM2_ESM.docx]

**Supplementary data**

**Figure S1:** Study flow chart


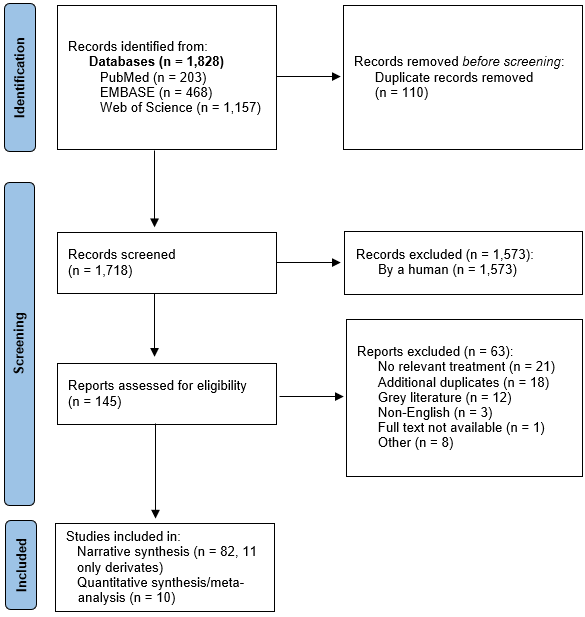


**Figure S2:**

**
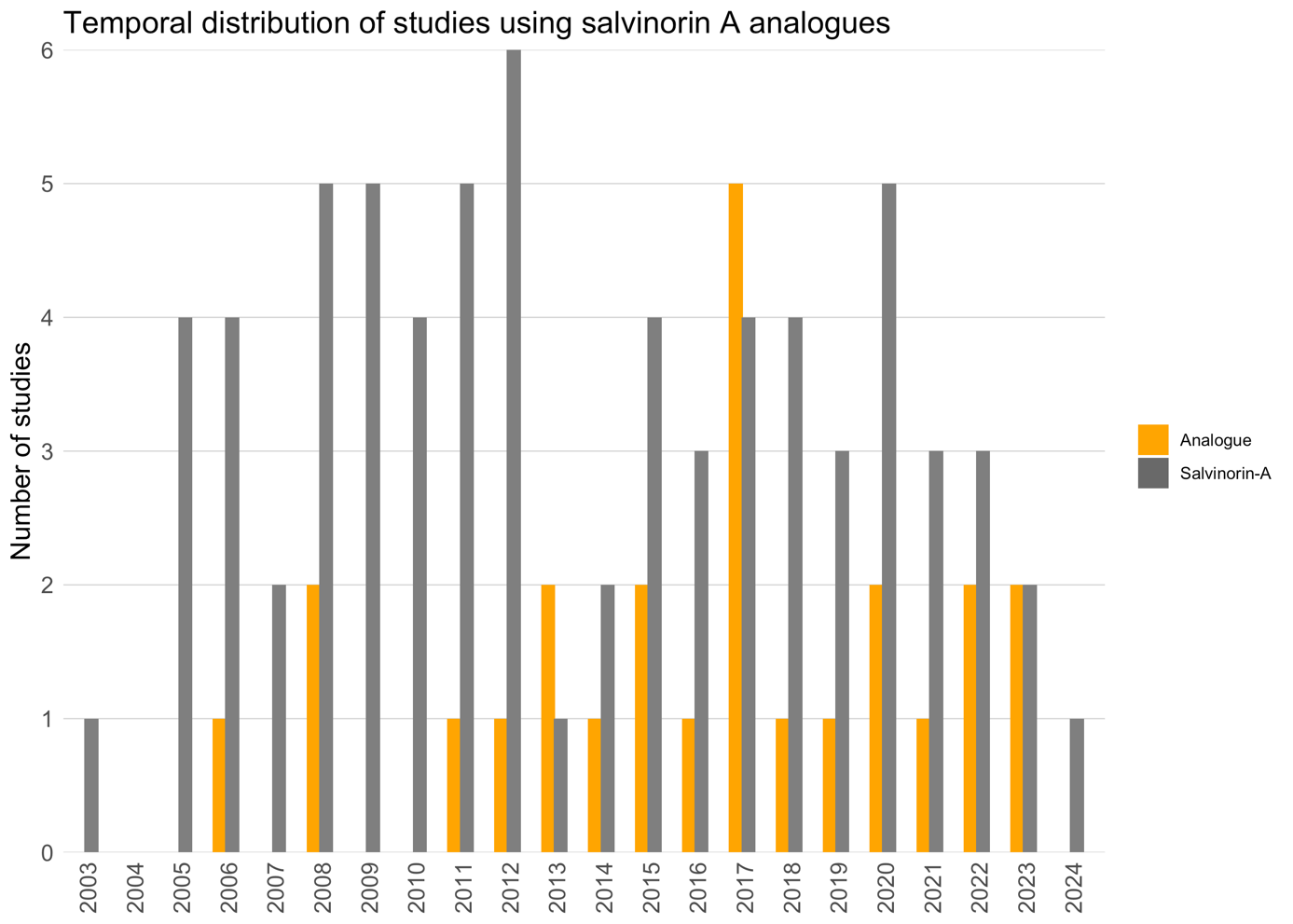
**

Number of studies per year assessing the therapeutic potential of salvinorin A or an analogue of salvinorin A. Studies using both salvinorin A and an analogue of salvinorin A were counted in each category.

**Table S1**: Search string

| salvinorin OR "salvia divinorum" OR "ska maría pastora" OR "seer's sage" OR "yerba de la pastora" OR "magic mint" |
| --- |

**Table S2:** Risk of bias assessment of the included studies.

| **Study_ID** | **Randomization** | **Blinding** | **Animal welfare** | **Sample size**  **Calculation** | **Conflict of**  **Interest** | **ARRIVE** |
| --- | --- | --- | --- | --- | --- | --- |
| Akins_2022 | 1 | 1 | 1 | 2 | 1 | 2 |
| Ansonoff_2006 | 2 | 2 | 1 | 2 | 1 | 2 |
| Aviello_2011 | 1 | 2 | 1 | 2 | 1 | 2 |
| Béguin_2008 | 2 | 2 | 1 | 2 | 1 | 2 |
| Braida_2007 | 2 | 2 | 1 | 2 | 2 | 2 |
| Braida_2008 | 1 | 2 | 1 | 2 | 1 | 2 |
| Braida_2009 | 1 | 2 | 1 | 2 | 1 | 2 |
| Braida_2011 | 1 | 2 | 1 | 2 | 1 | 2 |
| Butelman_2007 | 2 | 2 | 1 | 2 | 1 | 2 |
| Butelman_2009 | 1 | 1 | 1 | 2 | 2 | 2 |
| Butelman_2019 | 2 | 1 | 1 | 2 | 2 | 2 |
| Carlezon_2006 | 2 | 2 | 1 | 2 | 2 | 2 |
| Chartoff_2008 | 2 | 1 | 1 | 2 | 1 | 2 |
| Chartoff_2016 | 1 | 2 | 1 | 2 | 1 | 2 |
| Chen_2014 | 1 | 1 | 1 | 2 | 1 | 2 |
| Chen_2016 | 1 | 1 | 1 | 2 | 1 | 2 |
| Coffeen_2018 | 2 | 2 | 1 | 2 | 1 | 2 |
| Crowley_2016 | 2 | 2 | 2 | 2 | 2 | 2 |
| Crowley_2020 | 2 | 2 | 1 | 2 | 1 | 2 |
| Dong_2018 | 2 | 1 | 1 | 2 | 2 | 2 |
| Dong_2019 | 2 | 1 | 1 | 2 | 1 | 2 |
| Ebner_2010 | 1 | 2 | 1 | 2 | 2 | 2 |
| Ewald_2017 | 2 | 1 | 1 | 2 | 1 | 2 |
| Fajemiroye_2017 | 1 | 2 | 1 | 2 | 1 | 2 |
| Fantegrossi_2005 | 2 | 2 | 1 | 2 | 2 | 2 |
| Freeman_2014 | 1 | 2 | 1 | 2 | 2 | 2 |
| Guida_2012 | 2 | 1 | 1 | 2 | 1 | 2 |
| Harden_2012 | 1 | 2 | 1 | 2 | 1 | 2 |
| Hooker_2008 | 2 | 2 | 2 | 2 | 2 | 2 |
| Hooker_2009 | 2 | 2 | 1 | 2 | 2 | 2 |
| Huskinson_2020 | 2 | 1 | 1 | 2 | 1 | 2 |
| Jennings_2022 | 2 | 1 | 1 | 2 | 1 | 2 |
| Ji_2013 | 2 | 2 | 1 | 2 | 2 | 2 |
| John_2006 | 2 | 2 | 1 | 2 | 2 | 2 |
| Keasling_2019 | 2 | 2 | 1 | 2 | 1 | 2 |
| Kivell_2018 | 2 | 1 | 1 | 2 | 1 | 2 |
| Lamb_2012 | 2 | 1 | 1 | 2 | 1 | 2 |
| Mascarenhas_2020 | 2 | 1 | 1 | 2 | 1 | 2 |
| McCurdy_2006 | 2 | 2 | 2 | 2 | 2 | 2 |
| Misilimu_2022 | 1 | 1 | 1 | 2 | 1 | 1 |
| Morani_2009 | 2 | 2 | 1 | 2 | 2 | 2 |
| Morani_2012 | 1 | 1 | 1 | 2 | 1 | 2 |
| Morani_2013 | 2 | 1 | 1 | 2 | 2 | 2 |
| Moreira_2023 | 1 | 2 | 1 | 2 | 1 | 2 |
| Mowry_2003 | 2 | 2 | 2 | 2 | 2 | 2 |
| Negus_2012 | 2 | 2 | 1 | 2 | 2 | 2 |
| Nemeth_2010 | 2 | 2 | 1 | 2 | 2 | 2 |
| Paton_2017 | 2 | 1 | 1 | 2 | 1 | 2 |
| Paton_2020 | 2 | 1 | 1 | 2 | 2 | 2 |
| Paton_2021 | 2 | 1 | 1 | 2 | 1 | 2 |
| Paton_2022 | 2 | 1 | 1 | 2 | 1 | 2 |
| Potter_2011 | 2 | 2 | 1 | 2 | 1 | 2 |
| Prevatt_2011 | 2 | 2 | 2 | 2 | 2 | 2 |
| Riley_2014 | 2 | 2 | 1 | 2 | 1 | 2 |
| Schmidt_2005 | 2 | 2 | 2 | 2 | 2 | 2 |
| Serra_2015 | 2 | 2 | 1 | 2 | 2 | 2 |
| Sherwood_2017 | 2 | 1 | 1 | 2 | 2 | 2 |
| Simonson_2015 | 2 | 2 | 1 | 2 | 1 | 1 |
| Socala_2020 | 2 | 2 | 1 | 2 | 1 | 2 |
| Someya_2017 | 2 | 2 | 1 | 2 | 2 | 2 |
| Su_2011 | 2 | 2 | 1 | 2 | 2 | 2 |
| Su_2012 | 2 | 2 | 1 | 2 | 2 | 2 |
| Sufka_2013 | 2 | 2 | 1 | 2 | 2 | 2 |
| Sun_2018 | 1 | 2 | 1 | 2 | 1 | 2 |
| Sun_2019 | 1 | 2 | 1 | 2 | 1 | 2 |
| Tejeda_2010 | 2 | 2 | 1 | 2 | 2 | 2 |
| Teksin_2009 | 2 | 2 | 1 | 2 | 2 | 2 |
| Twardowschy_2015 | 2 | 2 | 1 | 2 | 1 | 2 |
| vandeWetering_2023 | 2 | 1 | 1 | 2 | 1 | 2 |
| Vazquez_2021 | 1 | 2 | 1 | 2 | 2 | 2 |
| Vazquez_2023 | 1 | 0 | 1 | 2 | 1 | 2 |
| Walentiny_2010 | 2 | 2 | 1 | 2 | 2 | 2 |
| Wang_2005 | 2 | 2 | 1 | 2 | 2 | 2 |
| Wang_2008 | 2 | 2 | 1 | 2 | 2 | 2 |
| Wang_2012 | 2 | 2 | 1 | 2 | 2 | 2 |
| White_2015 | 2 | 2 | 1 | 2 | 2 | 2 |
| Wu_2021 | 1 | 1 | 1 | 1 | 1 | 1 |
| Xin_2016 | 1 | 1 | 1 | 2 | 1 | 2 |
| Xin_2021 | 1 | 2 | 1 | 2 | 1 | 2 |
| Zamarripa_2020 | 1 | 2 | 1 | 2 | 1 | 2 |
| Zamarripa_2024 | 1 | 2 | 1 | 2 | 1 | 2 |
| Zhang_2005 | 1 | 2 | 1 | 2 | 2 | 2 |

Risk of bias was assessed using a pre-defined 6-item checklist: a.) Reporting of randomization b.) Reporting of blinding, c.) Reporting of an animal welfare statement, d.) Statement of a potential conflict of interest, e.) Sample size calculations provided, and f.) In accordance with the ARRIVE guidelines. 0=no, 1=yes, 2=Not reported. Studies are listed in alphabetical order.

**Table S3:** Studies assessing the effects of salvinorin A in animal models of addiction

| **Study**  (n = 11) | **Species** | **Salvinorin A dose(s)** | **Tested substance** | **Outcome measures** | **Effect of salvinorin A** |
| --- | --- | --- | --- | --- | --- |
| Chartoff et al., 2008 | Rats | 2 mg/kg (i.p.) | Cocaine | Locomotor activity tests, Immunohistochemistry | **Mixed effects:** Single acute administration of salvinorin A decreased behavioural and molecular responses to cocaine. Repeated administration (one administration per day on 6 of 8 days) potentiated locomotor and molecular responses to cocaine in a context-dependent manner. |
| Chartoff et al., 2016 | Rats | 2 mg/kg (i.p.) | Cocaine | Intracranial self-stimulation, Fast-scan cyclic voltammetry | **Mixed effects:** Acute administration of cocaine potentiated cocaine-induced reward and dopamine release when administered within 1 hour of salvinorin A. Delayed administration (24 hours later) resulted in a decrease in cocaine-induced reward and dopamine release. |
| Ewald et al., 2017 | Rats | 0.3 mg/kg (i.v.) | Cocaine | Self-administration with extinction and reinstatement testing | **Anti-Addictive:** Attenuation of reinstatement of drug-seeking behaviour. |
| Freeman et al., 2014 | Rhesus monkey | 0.0003 - 0.01 mg/kg (i.v.) | Cocaine,  Remifentanil | Concurrent-Choice Schedule of Reinforcement | **Anti-Addictive:** Dose-dependent decrease in choice for cocaine and remifentanil options. |
| Morani et al., 2009 | Rats | 0.3, 1 mg/kg (i.p.) | Cocaine | Self-administration with extinction and reinstatement testing | **Anti-Addictive:** Reduction of cocaine-induced drug seeking behaviour, measured over 60 min following salvinorin A injection. |
| Morani et al., 2012 | Rats | 0.3 mg/kg (i.p.) | Cocaine | Cocaine Sensitization and Stereotypy | **Anti-Addictive:** Reduction of cocaine-induced behavioural sensitization, with a significant attenuation of locomotion observed at 5, 10, 15, and 20 min following salvinorin A injection. |
| Potter et al., 2011 | Rats | 2 mg/kg/d (i.p.) | Cocaine | Intracranial self-stimulation | **Anti-Addictive:** Repeated administration produced a net decrease in the reward-potentiating effects of cocaine. |
| Prevatt et al., 2011 | Rats | 0.3 mg/kg (i.p.) | Cocaine | Self-administration with extinction and reinstatement testing | **Anti-Addictive:** Reduction of cocaine-induced drug seeking behaviour, measured over 2 hours following salvinorin A injection. |
| Van de Wetering et al., 2023 | Rats | 0.3, 2 mg/kg (i.p.) | Cocaine | Self-administration with extinction and reinstatement testing, Progressive Ratio Schedule of Reinforcement | **Anti-Addictive:** Reduction of cocaine-induced drug-seeking behaviour. No significant effect at attenuating the reinforcing effects of cocaine on a progressive ratio schedule. |
| Zamarripa et al.,2020 | Rhesus monkey | 0.006, 0.012 mg/kg/injection (i.v.) | Oxycodone | Progressive Ratio Schedule of Reinforcement | **Anti-Addictive:** Reduction of oxycodone self-administration. |
| Zamarripa et al.,2024 | Rhesus monkey | 1.0 - 3.2 μg/kg/injection (i.v.) | Cocaine,  Oxycodone | Concurrent-Choice Schedule of Reinforcement | **Anti-Addictive:** Reduction of cocaine and oxycodone self-administration in a dose-dependent manner. |

*Abbreviations: i.p., intraperitoneal; i.v., intravenous.*

**Table S4:** Studies assessing the effects of salvinorin A in animal models of pain

| **Study**  (n = 21) | **Species** | **Salvinorin A dose(s)** | **Outcome measures** | **Effect of salvinorin A** |
| --- | --- | --- | --- | --- |
| Akins et al., 2022 | Mice | 2.5 mg/kg (i.p.) | Hot plate test, Tail flick test | **Ineffective:** Single acute administration led to no significant antinociception. |
| Ansonoff et al., 2006 | Mice | 0.001 - 0. 3 mg (i.c.v.) | Tail flick test | **Antinociceptive:** Antinociception lasting for at least 45 minutes after single i.c.v. injection of 50 μg salvinorin A. |
| Aviello et al., 2011 | Mice | 0.5 - 2 mg/kg (i.p.) | LPS- and carrageenan-induced paw oedema, Formalin test | **Antinociceptive and anti-inflammatory:** Pretreatment with single shot of salvinorin A significantly reduced LPS- and carrageenan-induced paw oedema and formalin-induced inflammatory pain. |
| Coffeen et al., 2018 | Rats | 11.55 nm/ lL, 2 lL/min (Injection directly in the insular cortex) | Plantar test in a neuropathic pain model induced by the sciatic nerve ligature | **Antinociceptive:** Delayed response to thermal and mechanical nociceptive stimulation immediately after and 30 minutes after the microinjection of salvinorin A. |
| Ewald et al., 2017 | Mice | 0.1 - 12.5 mg/kg (s.c.) | Hot water tail withdrawal test | **Antinociceptive:** Cumulative doses of salvinorin A (0.1–12.5 mg/kg, administered every 30 min) produced antinociception, with a maximum possible effect of up to 18%, recorded 30 min after each dose. |
| Guida et al., 2012 | Mice | 0.5 - 2 mg/kg (i.p.) | Mechanical paw withdrawal threshold after mechanical pain stimulus, Electrophysiology, Immunohistochemistry, Immunoblotting | **Anti-allodynic and anti-inflammatory:** Repeated treatment of salvinorin A reduced mechanical allodynia, spinal hyperactivity, and glial activation, with modulation of inflammatory responses. |
| Jennings et al., 2022 | Rats | 0.0001 mg (i.pl.) | Paw withdrawal latency in a PGE2-stimulated heat allodynia assay | **Anti-allodynic:** Reduced PGE2-stimulated heat allodynia in aged rats, but not in young rats. Pre-treatment with arachidonic acid restored efficacy in young rats. |
| John et al., 2006 | Mice | 11.6 - 23.1 nmol (i.t.) | Tail flick test | **Antinociceptive:** Increased tail-flick latency in a dose-dependent manner. Significant effects were observed at 5, 10, and 15 min following the highest dose. |
| Kivell et al., 2018 | Mice | 1, 2 mg/kg (i.p.) | Hot water tail withdrawal test, Formalin test | **Antinociceptive and anti-inflammatory:** Increased thermal antinociception in the warm-water tail withdrawal test and attenuated phase 1 (nociceptive) and phase 2 (inflammatory) pain responses in the formalin test, while also significantly reducing formaldehyde-induced paw oedema. |
| Mascarenhas et al., 2020 | Rats | 0 - 0.45 mg/kg (i.p.) | Thermal plantar test, Formalin test | **Limited antinociceptive effect:** Salvinorin A did not show an effect in the thermal plantar test, tested 10-12 min after single injection. In the formalin test, it reduced pain only in 21-day-old subjects, but not in 7-day-old subjects, with observations conducted for 45 min after injection. |
| McCurdy et al., 2006 | Mice | 0.5 - 4 mg/kg (i.p.) | Hot plate test, Tail flick test, Writhing test | **Antinociceptive:** Dose-dependent antinociception that peaked at 10 minutes post-injection but rapidly returned to baseline. |
| Negus et al., 2012 | Rats | 0.1 - 3.2 mg/kg (i.p.) | Lactic acid-stimulated stretching assay | **Antinociceptive:** Dose- and time-dependent reduction of acid-stimulated stretching, significant at 1.0 and 3.2 mg/kg (not 0.1 or 0.32 mg/kg) and at 10 and 30 min (not 100 min) post-administration. |
| Paton et al., 2017 | Mice | 1, 2 mg/kg (i.p.) | Hot water tail withdrawal test, Formalin test, Histology, Flow cytometry, Mechanical and cold allodynia via Paclitaxel-induced neuropathic pain model | **Antinociceptive and anti-inflammatory:** Analgesic effects in the tail-withdrawal and formalin assays. Reduced oedema and decreased neutrophil infiltration into inflamed tissue. Suppressed mechanical and cold allodynia in paclitaxel-induced neuropathic pain. |
| Paton et al., 2020 | Mice | 2 mg/kg (i.p., s.c.) | Hot plate test, Formalin test | **Antinociceptive and anti-inflammatory:**  Significant antinociceptive effects at 15 and 30 min post-treatment in the hot plate test. In the formalin test, significant reduction of both nociceptive and inflammatory pain-related behaviours. |
| Sherwood et al., 2017 | Mice | 2 mg/kg (i.p.) | Hot water tail withdrawal test, Formalin test | **Antinociceptive and anti-inflammatory:** Significant analgesic effects seen at 5 and 10 min, respectively, and lasting until 30 min in the warm-water tail withdrawal test. Significant attenuation in both phase 1 (nociceptive) and phase 2 (inflammatory) pain responses in the formalin test. |
| Simonson et al., 2015 | Rats | 1 mg/kg (i.p.) | Hot water tail withdrawal test | **Antinociceptive:** Significant effects at 5, 10 and 15 min after single drug administration. |
| Van de Wetering et al., 2023 | Mice | 1 mg/kg (i.p.) | Hot water tail withdrawal test | **Antinociceptive:** Significant antinociceptive effects primarily within the first 15 min after administration of a single dose of salvinorin A. |
| Walentiny et al., 2010 | Mice | 1 - 10 mg/kg (i.v.) | Tail flick test | **Antinociceptive:** Demonstrated dose-dependent antinociceptive activity, with 95% maximum possible effect observed at 10 mg/kg. |
| Wang et al., 2005 | Mice | 15 - 50 mg/kg (s.c.) | Writhing test | **Ineffective:** No dose-related effects were observed. The maximum mean percent antinociception was only 16.5% (with the 50 mg/kg dose). Salvinorin A was administered as a single injection 20 min before i.p. acetic acid, and responses were recorded for 10 min. |
| Wang et al., 2008 | Rats | 10 mg/kg (i.p.) | Hot plate test | **Ineffective:** No significant antinociceptive effect in the hot plate test 30 min after administration of single dose. |
| White et al., 2015 | Mice | 3 mg/kg (s.c.) | Hot plate test | **Antinociceptive:** Significantly delayed pain response 10 minutes posttreatment. |

*Abbreviations: i.p., intraperitoneal; i.c.v., intracerebroventricular; s.c., subcutaneous; i.pl., intraplantar; i.t., intrathecally; i.v., intravenous.*

**Table S5:** Studies assessing the effects of salvinorin A in animal models of depression

| **Study**  (n = 9) | **Species** | **Salvinorin A dose(s)** | **Outcome measures** | **Effect of salvinorin A** |
| --- | --- | --- | --- | --- |
| Béguin et al., 2008 | Rats | 0.125 - 4 mg/kg (i.p., p.o.) | Intracranial Self-Stimulation | **Depressogenic:** Dose-dependently elevated brain reward thresholds in the ICSS test, with oral administration showing diminished effects compared to intraperitoneal administration. |
| Braida et al., 2007 | Zebrafish | 0.1 - 10 μg/kg (i.m.) | Swimming activity via squared observational chamber | **Mixed effects:** Dose-dependently increased swimming at lower doses (0.1–0.2 μg/kg), while inducing trance-like behaviour at higher doses (5–10 μg/kg). Salvinorin A showed both stimulating effects at 0.2 μg/kg and depressive effects at 10 μg/kg, with fish observed for 30 seconds immediately after injection and every 5 minutes for a total of 30 minutes. |
| Braida et al., 2009 | Mice, Rats | 0.001 - 1000 μg/kg (s.c.) | Forced swim test, Tail suspension test | **Antidepressant:** Dose-dependently decreased immobility and increased swimming in the FST, and reduced immobility in the TST, with observations conducted 20 minutes post-treatment over a 5–6-minute period. |
| Butelman et al., 2019 | Mice | 0.56 - 1.8 mg/kg (i.p.) | Self-grooming behaviour via the splash test | **Depressogenic:** Dose-dependent decrease in self-grooming, a marker of anhedonia, observed for 5 minutes at 15 minutes post-treatment. |
| Carlezon et al., 2006 | Rats | 0.125 - 2 mg/kg (i.p.) | Forced swim test, Intracranial Self-Stimulation, In Vivo Microdialysis | **Depressogenic:** Increased immobility in the FST following three treatment injections at 23, 5, and 1 hour before a 5-minute observation period. Effective doses did not impair locomotion in an open field test. Additionally, salvinorin A elevated ICSS thresholds and decreased extracellular dopamine in the NAc. |
| Ebner et al., 2010 | Rats | 0.25, 2 mg/kg (i.p.) | Electrically evoked phasic dopamine release in the NAc core or shell using fast scan cyclic voltammetry, Intracranial Self-Stimulation, Progressive ratio responding for sucrose | **Depressogenic:** Decrease of phasic dopamine release in the NAc core and shell, increased ICSS thresholds, and reduced sucrose-reinforced PR responding, indicating decreased motivation. |
| Fajemiroye et al., 2017 | Mice | 5 - 20 mg/kg (p.o.) | Irwin test, Open field test, Forced swim test, Tail suspension test | **No effect:** No significant alterations in respective measures of the Irwin test, open field test, tail suspension test, and forced swim test. |
| Harden et al., 2012 | Rats | 1 mg/kg (i.p.) | Sucrose preference test | **Antidepressant:** Chronic three-week administration reversed anhedonia in rats exposed to chronic mild stress. Weekly assessments were conducted more than 4 hours post-treatment to avoid acute drug effects. |
| Morani et al., 2012 | Rats | 0.3 mg/kg (i.p.) | Forced swim test | **Depressogenic:** Decreased climbing and swimming time with increased immobility in the FST, observed for 5 minutes at 5 minutes post-treatment. The same dose did not affect spontaneous locomotion in the open field test. |

*Abbreviations: i.p., intraperitoneal; p.o., peroral; s.c., subcutaneous.*

**Table S6:** Studies assessing the effects of salvinorin A in animal models of cerebrovascular diseases

| **Study** (n=13) | **Species** | **Dose(s);**  **Time of administration** | **Disease model** | **Outcome measures** | **Effect of salvinorin A** |
| --- | --- | --- | --- | --- | --- |
| Chen et al., 2014 | Neonatal mice | 0.5 mg/kg (i.p.); immediately before hypoxia | **Hypoxia** via hypoxic gas exposure for 120 min | Mortality rate, Weight, Developmental motor behaviours, Placing reflex, Cliff aversion, Negative geotaxis test, Righting response and forelimb grasping tests, Neurodevelopmental milestones, Long-term neurobehavioral effects.  Analyses were conducted daily from postnatal day 2 to postnatal day 21, and at the age of 10-11 weeks. | **Neuroprotective and improved survival:** Decreased mortality from 70% to 38.5%, improved body weight, earlier emergence of rearing and rescued delays in forelimb grasping, cliff aversion, righting response, eye opening, and rearing activity. No significant long-term neurological differences beyond 21 days. |
| Chen et al., 2016 | Mice | 12.5 - 50 μg/kg (i.n.); 10 min after initiation of reperfusion | **Cerebral ischemia** via MCAO for 120 min | Neurobehavioral outcomes, Infarct volume, Blood-brain barrier function, Immunohistochemistry, Immunoblotting.  Analyses were conducted 24 h after reperfusion. | **Neuroprotective:** Reduced neurological deficit, infarct size, blood-brain barrier impairment, apoptosis, and inflammation. |
| Dong et al., 2018 | Rats | 10, 20 μg/kg (i.v.); 10 min after occlusion together with reperfusion | **Forebrain ischemia** via BCCAO for 10 min | Neurological outcomes, Histology, Biochemical Assays (Western blotting), Dilation response of pial arteries to hypotension and hypercapnia.  Analyses were conducted 1 h (pial artery regulation), 24 h (western blot), and 1 d, 2 d, and 5 d (motor function) post-ischemia. | **Neuroprotective and vasoprotective:** Preservation of pial artery autoregulation to hypotension and hypercapnia after ischemia via PI3K/Akt/cGMP pathway, with improved motor function and reduced cortical necrosis and apoptosis. |
| Dong et al., 2019 | Rats | 20 μg/kg (i.v.); after reperfusion | **Cerebral ischemia** via MCAO for 90 min | Neurological scores, Infarct volume, Cerebral oedema, Blood-brain barrier function, Immunofluorescence.  Analyses were conducted 24 h (infarct size, brain oedema), 50 h (blood-brain barrier), and 1 d, 3 d, 5 d, and 14 d (neurological function) post-ischemia. | **Neuroprotective:** Reduced neurological deficit, infarct size, brain oedema and blood-brain barrier impairment. |
| Misilimu et al., 2022 | Mice | 50 μg/kg (i.n.); once every 2 d for 6 d, starting 10 min after reperfusion | **Ischemic stroke** via tMCAO for 60 min | Sensory and motor function, Neurocognitive function, Blood-brain barrier function, Immunofluorescence.  Analyses of sensory, motor, and cognitive function were conducted 3, 5, 7, 14, 21, 28, 30, and 35 d post-ischemia. | **Neuroprotective:** Reduced pro-inflammatory factors, macrophage infiltration, and brain atrophy, while improving sensory, motor, cognitive functions, and blood-brain barrier preservation. |
| Su et al., 2011 | Piglets | 10 nM and 1 μM dissolved with alcohol (NA) | **Cerebral vasoconstriction** induced via hypocapnia and endothelin. | Pial artery diameter monitoring | **Vasoprotective:** Cerebrovascular dilation via κ-opioid receptor, NOS activation, and ATP-sensitive potassium channels, with sustained effects under resting tone, hypocarbia, and endothelin-induced constriction. Dilation was observed immediately after salvinorin A administration and lasted less than 5 min. When administered every 2 min, sustained dilation effects were observed for 30 min. |
| Su et al., 2012 | Piglets | 10 μg/kg (i.v.); 30min before hypoxia/ischemia | **Hypoxia** via nitrogen ventilation for 10 min, followed by **cerebral ischemia** via saline infusion for 20 min | Dilation response of pial arteries to hypotension and hypercapnia | **Vasoprotective:** Preservation of pial artery autoregulation to hypotension and hypercapnia after hypoxia and global cerebral ischemia. |
| Sun et al., 2018 | Rats | 2, 10 μg/kg (i.p.); 24, 48, and 72 h after SA | **CVS** after **SAH** via endovascular perforation | Neurological scores, Histology, Imaging (MRI), Biochemical Assays (ELISA, Western blotting).  Analyses were conducted 12, 24, 48, and 72 h (neurological scores), and 72 h (imaging) after SAH. | **Neuroprotective:** Alleviation of CVS after SAH through increased eNOS and NO levels, decreased ET-1 and AQP-4 expression, and improved neurological scores, histological findings, and MRI outcomes. |
| Sun et al., 2019 | Rats | 10 μg/kg (i.p.); 24, 48, and 72 h after SA | **Early brain injury** after **SAH** via endovascular perforation | Imaging (MRI), Histology, Biochemical Assays (Western blotting).  Analyses were conducted 72 h after SAH. | **Neuroprotective:** Reduction of neuronal apoptosis and inflammation in the hippocampus after SAH via PI3K/Akt pathway activation, alongside decreased expression of apoptotic and inflammatory markers. |
| Wang et al., 2012 | Piglets | 10 μg/kg (i.v.); 0 and 30 min after hypoxia/ischemia | **Hypoxia** via nitrogen ventilation for 10 min, followed by **cerebral ischemia** via saline infusion for 20 min | Measurement of pial artery autoregulation in response to hypercapnia and hypotension.  Analyses were conducted 1 h after hypoxia/reperfusion. | **Vasoprotective:** Preservation of pial artery autoregulation to hypercapnia and hypotension via κ-opioid receptor and ERK pathway. |
| Wu et al., 2021 | Rhesus monkey | 25 μg/kg (i.n.); 20 min after occlusion | **Ischemia** via middle cerebral artery M2 segment occlusion with an autologous blood clot | Neurological function, Infarct volume, Imaging (MRI).  Analyses were conducted 24 h, and 28 d (imaging), and 1, 2, 3, 7, 14, and 28 d (neurological function) post-ischemia. | **Neuroprotective:** Reduction of infarct volume and improvement of neurological function. Enhanced MRI imaging showed reduced occupancy effect and diffusion limitation in lesions. |
| Xin et al., 2016 | Rats | 10 μg/kg (i.v.); 10 min after occlusion | **Ischemia** and **Reperfusion injury** via BCCAO for 10 min | Neuromotor and neurocognitive tests, Cerebral oedema, Histology, Immunoblotting, Immunohistochemistry.  Analyses were conducted 24 h (histology, immunoblotting, immunohistochemistry), and 1, 2, and 5 d (neurological function) post-ischemia. | **Neuroprotective:** Reduced brain oedema and apoptosis in the hippocampus, cortex, and striatum, with partial recovery of motor and cognitive function. |
| Xin et al., 2021 | Rats | 20 μg/kg (i.v.); 60 min after occlusion | **Ischemia** and **Reperfusion injury** via MCAO for 60 min | Neurological scores, Infarct volume, Cerebral oedema, Blood-brain barrier function.  Analyses were conducted 24 h (infarct volume, cerebral oedema, blood-brain barrier function), and 1, 2, and 5 d (neurological function) post-ischemia. | **Neuroprotective:** Alleviation of cerebral vascular injury, infarct volume, cerebral oedema, and blood-brain barrier permeability impairment, with improved neurological scores. |

*Abbreviations: i.p., intraperitoneal; i.n., intranasal; i.v., intravenous; MCAO, middle cerebral artery occlusion, BCCAO, Bilateral common carotid artery occlusion; tMCAO, transient middle cerebral artery occlusion; CVS, Cerebral vasospasm; SAH, subarachnoid haemorrhage; KOR, κ-opioid receptor.*
